# Biological Management of Banana Fusarium Wilt Caused by *Fusarium oxysporum* f. sp. *cubense* Tropical Race 4 Using Antagonistic Fungal Isolate CSR-T-3 (*Trichoderma reesei*)

**DOI:** 10.3389/fmicb.2020.595845

**Published:** 2020-12-16

**Authors:** Thukkaram Damodaran, Shailendra Rajan, Manoharan Muthukumar, Ram Gopal, Kavita Yadav, Sandeep Kumar, Israr Ahmad, Nidhi Kumari, Vinay K. Mishra, Sunil K. Jha

**Affiliations:** ^1^Indian Council of Agricultural Research-Central Soil Salinity Research Institute, Regional Research Station, Lucknow, India; ^2^Indian Council of Agricultural Research-Central Institute for Subtropical Horticulture, Lucknow, India

**Keywords:** *Trichoderma reesei*, banana, Fusarium wilt TR4, antifungal, LC-MS, gene expression, field evaluation

## Abstract

*Fusarium* wilt in bananas is one of the most devastating diseases that poses a serious threat to the banana industry globally. With no effective control measures available to date, biological control has been explored to restrict the spread and manage the outbreak. We studied the effective biological control potential of different *Trichoderma* spp. in the management of *Fusarium oxysporum* f. sp. *cubense* tropical race 4 (Foc TR4). Expression of the defense related genes and metabolites in banana plants inoculated with Foc TR4 and treated with effective *Trichoderma* sp interactions were also studied. The *in vitro* growth inhibition of Foc TR4 by *Trichoderma reesei* isolate CSR-T-3 was 85.19% indicating a higher antagonistic potential than other *Trichoderma* isolates used in the study. Further, in *in vivo* assays, the banana plants treated with the isolate CSR-T-3 *T. reesei* had a significant reduction in the disease severity index (0.75) and also had increased phenological indices with respect to Foc TR4 treated plants. Enhanced activity of defense enzymes, such as β-1, 3-glucanase, peroxidase, chitinase, polyphenol oxidase, and phenylalanine ammonia lyase with higher phenol contents were found in the *Trichoderma* isolate CSR-T-3 treated banana plants challenge-inoculated with Foc TR4. Fusarium toxins, such as fusaristatin A, fusarin C, chlamydosporal, and beauveric acid were identified by LC-MS in Foc TR4-infected banana plants while high intensity production of antifungal compounds, such as ß-caryophyllene, catechin-o-gallate, soyasapogenol rhamnosyl glucoronide, peptaibols, fenigycin, iturin C19, anthocyanin, and gallocatechin-o-gallate were detected in *T. reesei* isolate CSR-T-3 treated plants previously inoculated with Foc TR4. Gene expression analysis indicated the upregulation of *TrCBH1*/*TrCBH2, TrXYL1, TrEGL1, TrTMK1, TrTGA1*, and *TrVEL1* genes in CSR-T-3 treatment. LC-MS and gene expression analysis could ascertain the upregulation of genes involved in mycoparasitism and the signal transduction pathway leading to secondary metabolite production under CSR-T-3 treatment. The plants in the field study showed a reduced disease severity index (1.14) with high phenological growth and yield indices when treated with *T. reesei* isolate CSR-T-3 formulation. We report here an effective biocontrol-based management technological transformation from lab to the field for successful control of *Fusarium* wilt disease caused by Foc TR4 in bananas.

## Introduction

Fusarium wilt caused by *Fusarium oxysporum* f. sp. *cubense* tropical race 4 (Foc TR4) is considered to be one of the most devastating diseases of bananas limiting banana production worldwide (Ploetz and Pegg, 2000; Dita et al., 2018). The pathogen was first reported in South East Asia during the early 1990s and toward the end of the century, endemic outbreak of Foc TR4 was realized over large areas in the northern territory of Australia, China, Indonesia, Malaysia, and Taiwan (Ploetz, 2006a,b). Most recently, severe outbreak of Foc TR4 in the northern region in India covering the states of Uttar Pradesh and Bihar threatening banana cultivation in the region were reported, which is the largest producer of bananas (Damodaran, 2018). The polycyclic nature of the disease suggests that its multiple cycle of infection in bananas is causing devastating losses over a period of time (Buddenhagen, 2009). Different types of toxins are produced by Fusarium which are involved in pathogenesis and disease development. The major toxins that are reported from Fusarium species include; nivalenol, zearalenone, fumonisins, trichothecene, fusarins, and Fusaric acid (Niehaus et al., 2014; Dong et al., 2017; López-Díaz et al., 2017; Perincherry et al., 2019; Qiu et al., 2019). Differential colonization patterns of Foc race 4 from race 1 were identified and explained by upregulation of pathogenicity-associated genes, such as *Fga1, Fhk1, Fow2*, and *Ste12* that allowed faster colonization in the xylem with higher virulence (Guo et al., 2015). The discrimination of Foc TR4 from Foc race 1 proved very efficient with the PCR-based detection employed for secreted in xylem (*SIX*) genes (Carvalhais et al., 2019) and comparative analysis of various specific primers has also ascertained the efficiency of *SIX* gene-specific primers in the molecular detection of Foc TR4 (Magdama et al., 2019). These advances have now paved the way for a reliable dependable confirmatory assay for the early detection/diagnosis of Foc TR4.

Management and control of Foc TR4 is a matter of serious concern and different approaches, such as chemical and biological control, manipulation of cultural practices, and resistant breeding have been attempted by different research groups. The ability of Foc TR4 to survive in the absence of its host is a significant factor limiting the successful management of this disease through agronomic practices like crop rotation (Brandes, 1919). Besides this, use of chemical control measures involving corm injection and soil drenching of carbendazim fungicide failed to provide sustainable management of the disease but their repeated use has raised environmental concerns (Getha and Vikineswary, 2002). Application of soil fumigants, field sanitation, and flood fallowing provided limited control over a large area (Ploetz et al., 1990). In another approach using resistance breeding, some advances were reported for evolving resistant varieties and hybrids against Foc race 1 (Damodaran et al., 2009) but to date no successful reports are available for Foc TR4 (de Matos et al., 2011; Amorim et al., 2013; Garcez et al., 2016; Rebouças et al., 2018; Ribeiro et al., 2018; Gonçalves et al., 2019). Moreover, the selection of resistant cultivars seems to be an effective control strategy against the disease, however, the existence of poor fertility in the Cavendish group (AAA) limited its improvement programs through conventional breeding (Aguilar Moran, 2013). Thus, the only alternative approach for management is by exploring antagonistic and growth promoting fungi or bacteria which have earlier been successfully demonstrated in biological control of soil borne pathogens in different crop plants (Tan et al., 2015, Khan et al., 2018). Rhizospheric existence and endophytic colonization of the potential antagonist are known to not only suppress the pathogen but also incite systemic-induced host plant resistance (Ting et al., 2009, Sood et al., 2020).

*Trichoderma* (Teleomorph: *Hypocrea*) is a fast-growing soil inhabitant fungus known for the production of a large numbers of spores and has been reported to have biocontrol potentials (Monfil and Casas-Flores, 2014). The antagonism of *Trichoderma* against phytopathogens is achieved by mycoparasitism (Papavizas, 1985) through the production of enzymes (chitinases, glucanases, and proteases) that degrade the fungal cell wall which is mainly composed of chitin (Qian et al., 2019) as well as antibiosis (Harman and Kubicek, 1998); through production of secondary metabolites and antimicrobial compounds active against a wide array of phytopathogens (Speckbacher and Zeilinger, 2018). Additionally, when the plant encounters the pathogen it induces a host defense mechanism while on interaction with a non-pathogenic organism the mechanism for induced systemic resistance (ISR) gets activated (Hermosa et al., 2013; Mukherjee et al., 2013). A plethora of studies suggest that *Trichoderma* spp. induce systemic resistance to the subsequent attack by the pathogens in the host plants (Martínez-Medina et al., 2013). Elevated production of antioxidants like polyphenols, cell wall degrading enzymes like glucanases, chitinases, etc. by Trichoderma probably induced the host defense mechanism against the invading pathogens including Fusarium (Kavino et al., 2009). Though, long term efficacy of biocontrol agents in field studies have seldom come up with repeatability at a field level (Ploetz, 2004), yet, repeated application of biocontrol agents has resulted in the suppression of soil borne pathogens. These discoveries showed promise for exploring antagonistic biocontrol agents and developing a package for the management of the Foc TR4 pathogen causing Fusarium wilt in bananas. The prime focus of the study was to identify an elite antagonistic fungus by screening different *Trichoderma* isolates from diverse biotic and abiotic stressed environments that has the potential to inhibit the Foc TR4 pathogen, assess its efficacy in the management of the disease under pot culture and sick field experiments, and to decipher its antifungal mode of action/mechanism in disease suppression through gene expression and LC-MS analysis. We report here a systematic development of a technological innovation involving a *Trichoderma* isolate and its field transformation as a biocontrol agent toward successful management of banana wilt caused by *Fusarium oxysporum* f. sp. *cubense* tropical race 4.

## Materials and Methods

### Collections of *Trichoderma* spp. and *Fusarium oxysporum* f. sp. *cubense* Tropical Race 4

The virulent strain of the *Fusarium oxysporum* f. sp. *cubense* tropical race 4 was collected from the culture collections of the Soil Microbiology Laboratory, ICAR-Central Soil Salinity Research Institute, Regional Research Station, Lucknow, India that had submitted the first report on the outbreak of the disease in India (Damodaran et al., 2018). The three *Trichoderma* isolates used in the study are described as follows; (i) *Trichoderma reesei* (CSR-T-3, MK050013) isolated from the rhizosphere soil of banana cultivar G-9 grown in salt affected soil, (ii) *Trichoderma asperellum* (CSR-T-4, MN227242) isolated from the disease suppressive rhizosphere of banana cultivar G-9 grown in the Foc TR4 affected region of Uttar Pradesh, India, and (iii) *Trichoderma koningiopsis* (CSR-T-2, KJ812401) obtained from the rhizosphere of *Saccharam spontaneum* in the barren sodic soil of Uttar Pradesh, India. These isolates were identified and submitted to the culture collections (NBIMCC-SF-0030) at the ICAR-National Bureau of Agriculturally Important Microorganisms (ICAR-NBAIM), Mau, India and have been reported in our earlier studies (Damodaran et al., 2019c; Yadav et al., 2020).

### *In vitro* Antagonistic Assays

The three isolates of the *Trichoderma* species were evaluated for their *in vitro* antagonistic potential against CSR-F-1 (the virulent strain of Foc TR4) through a dual culture test as described by Tian et al. (2016). Mycelial disks of Foc TR4 and *Trichoderma* spp. from actively growing colonies were placed on a 9 cm diameter petri dish filled with potato dextrose medium (PDA) in combinations. The isolates were placed at 5.5 cm apart on the same plate and were incubated for 72 h at 22°C. Foc TR4 isolate CSR-F-1 was also placed without disks of *Trichoderma* isolates as control. The growth of the isolates was monitored and observations were recorded at an interval of every 24 h from which the percentage of pathogen growth inhibition was calculated. The inhibition efficacy of *Trichoderma* isolates on the mycelia growth of Foc TR4 and the radius of the Foc colony in each plate were measured, and a standard growth curve was derived as described by Matarese et al. (2012). The distance between the Foc TR4 inoculating point and furthest point of the colony in control plates (r1) and the distance between the Foc TR4 sowing point and the edge of the colony where the mycelia came in contact with *Trichoderma* spp. in the treatment plates was taken at an interval of 24 h for calculating the inhibition percentage (IP) using the formula; IP = ((r1 – r2)/r1)^*^100. This *in vitro* dual culture assay was carried out in five replicates and the data was statistically analyzed by analysis of variance (ANOVA) using Duncan's multiple range tests using the SAS 9.0 software.

### SEM Analysis of Dual Culture Interaction to Ascertain Antagonism of the Best Performing Isolate

Scanning electron microscopy of the dual culture regions was carried out for establishing and observing the CSR-T-3 antagonism against Foc TR4 (CSR-F-1) as described in protocol given by Kumar et al. (2018). Pieces of agar from the interaction zones of the dual culture plates at their early stage of interaction were fixed in 3% glutaraldehyde dissolved in 0.1 M phosphate buffer (pH 7.0). The specimens were dehydrated using acetone at a series of concentration (30, 50, 60, 70, 80, 90, and 95%) for 20 min. The samples were dried for 30 min and mounted on a steel stub with double sided carbon tape. The samples were finally coated with a film of gold-palladium alloy under vacuum and observed with a scanning electron microscope (SEM, Fei Quanta 200) at Bhaba Bhimrao Ambedkar University, Lucknow, India. The control plates involving the individual plates of *T. reesei* (CSR-T-3) and Foc TR4 (CSR-F-1) were also maintained for comparison.

### *In vivo* Assay of Antifungal Potential Using Pot Culture

The *in vivo* assays were performed with the best performing *Trichoderma* isolate from the *in vitro* dual culture assay. A systematic pot culture experiment was carried out in June-July 2017 and June-July 2018 at the ICAR-Central Soil Salinity Research Institute, Regional Research Station, Lucknow, India by following a completely randomized design (CRD) with three replicates. The temperature of the green house during the experimental period ranged from 29 to 32°C with 70% humidity. The fungal culture was prepared by inoculating the 5-days-old culture of the best performing *Trichoderma* isolate in potato dextrose broth (PDB) with subsequent incubation for 5 days at 28°C in an incubated shaker. The spore suspension was obtained by filtering the broth through double-layered cheese cloth. The filtrate thus obtained was diluted 50 times and the spore number was adjusted from 10^7^ to 10^8^ using a hemocytometer. About 100 ml of the diluted filtrate was added in sterile soil. Likewise, the pathogen inoculum was prepared using the same procedure and the spore count was adjusted from 10^5^ to 10^6^. For sterilizing the soil, normal clayey loam soil (pH 7.45, E.C. 0.32 dS/m, organic carbon content of 3.0 g/kg) was collected from the research farm of the Institute, passed through a 20 mm mesh sieve and sterilized in an autoclave at 121°C for 30 min for 3 consecutive days.

Secondary hardened tissue cultured banana plantlets of the Grand Naine variety (susceptible) of uniform height were selected to conduct the experiment. The plants were inoculated with the pathogen by dipping them in the pathogen spore suspension containing 10^6^ conidia/ml for 30 min as described by Pérez-Vicente et al. (2014) before planting. Three treatments viz, TC: uninoculated plants, TF: plants inoculated with CSR-F-1 (Foc TR4) at the time of planting and TFTR: plants inoculated with CSR-F-1 (Foc TR4) along with soil drenching with suspension of the best performing *Trichoderma* isolate were included in the pot experiment with five replicates for each treatment. The experiment was repeated three times.

The phenological indices of banana seedlings like plant height (cm), girth (cm), and number of leaves was noted at 0, 30, 60, and 90 days after planting. The morphological growth-related parameters like root biomass and shoot biomass were recorded at the end of the experiment. The wilt disease was scored by adopting the following disease scale as followed by Pérez-Vicente et al. (2014).

**Table:** 

Grade 1	No symptoms
Grade 2	10–20% initial corm discoloration
Grade 3	20–40% slight discoloration of the corm
Grade 4	40–60% discoloration of the corm
Grade 5	60–80% complete discoloration of the corm

$$\begin{array}{l} \text{Disease index }=\\ \frac{\text{£}\left(\text{Number of diseased plants in each grade}\times \text{value of relative grade}\right)}{\text{Total number inspected}\times \text{5}} \end{array}$$

Besides this, the chlorophyll content was also estimated at the end of the experiment. Chlorophyll a, b, and total chlorophyll contents were estimated by following the method of Sadasivam and Manickam (1996) using 80% acetone and magnesium carbonate. The data were statistically analyzed by subjecting them to analysis of variance (ANOVA) and means were compared by a Duncan's multiple range test (*p* < 0.05) (Rangaswamy, 1995).

### Liquid Chromatography Coupled With Mass Spectrometric (LC-MS) Analysis

LC-MS analysis was performed in the plant samples of treatments TC (treatment negative control), TF (treatment Foc-TR4), and TFTR (treatment Foc-TR4 and *Trichoderma reesei* isolate CSR-T-3) of the pot experiment to identify important organic compounds and their role in inducing host tolerance. The whole plant was uprooted, washed with sterile distilled water, and then shade-dried for 30 days. Further the protocol for sample preparation was as prescribed by the Sophisticated Analytical Instrumentation Facility (SAIF), Central Drug Research Institute (CDRI), Lucknow, Uttar Pradesh, India.

A total of 250 g of dry weight plant material was used for the preparation of plant extracts consisting of root, leaves, and stem. Ethanol crude extract was obtained by crushing the plants in 70% ethanol which was further purified by using ethanol acetate. A small fraction of the solvent free plant extract was collected in Eppendorf for further analysis. The extract residue was stored at 4°C until used for the antimicrobial assay.

The ESI-LC–MS of the TLC purified sample was analyzed using a MICROMASS QUATTRO II triple quadrupole mass spectrometer with a JASCO PU-980 HPLC pump at the Sophisticated Analytical Instrumentation Facility, CSIR-Central Drug Research Institute, Lucknow, India. The WATER SPHERISORB ODS 2 (250 _ 4.6 mm _ 5 l) column was used with acetonitrile: water +0.1% formic acid solvent system. Gradient elution was performed at 1.0 ml/min. The photodiode array was monitored at 200–650 nm and recorded at 220 nm. The mass spectra were scanned in the range 80–1,000 DA in 2.5 s. The ESI capillary was set at 3.5 kV and the cone voltage at 40 V. The m/z spectral chromatographs were analyzed, key metabolites were predicted based on already published reports and tabulated for comparative analysis between the TC, TF, and TFTR samples.

### Biocontrol Mechanism of *Trichoderma reesei* Against Foc Through Gene Expression Analysis

To study the basis of resistance achieved in banana plants against Foc TR4 through drenching the soil with the best performing *Trichoderma* isolate at the time of planting, the expression levels of the genes of *T. reesei* and Foc TR4 were studied using a real time-polymerase chain reaction (RT-PCR) assay. Genomic sequence information for the genes involved in mycoparasitism *viz*., *TrCBH1*/*TrCBH2, TrXYL1*, and *TrEGL1* encoding cellobiohydrolase, xylanase, and endoglucanase, respectively and few genes involved in the signaling transduction pathway leading to secondary metabolite production in *T. reesei*, i.e., *TrTMK1, TrTGA1*, and *TrVEL1* genes encoding mitogen activated protein kinase (MAPK), G-protein alpha subunit, and VELVET1 protein, respectively were mined from the whole genome sequence of *T. reesei* QM6a as reported by Martínez-Hidalgo et al. (2015) (Bioproject: PRJNA15571) available at NCBI database. Similarly, the genes involved in Fusaric acid biosynthesis were mined from the whole genome information of *F. oxysporum* f. sp. *cubense* (Foc TR4) in the NCBI database (Bioproject: PRJNA529756). The selection of these genes was based on the well-established antagonistic biocontrol potential evincing molecular mechanism of *Trichoderma* sp. against *Fusarium* species earlier reported by Druzhinina et al. (2012) and Zeilinger et al. (2016). The primers for the genes used in RT-PCR analysis were designed for exonic segments contributing to the functional domains based on protein structural domain analysis and exon predictions (phylogenetic analysis of genes and structural predictions are presented in Supplementary Figures 1, 2) using the primer3 software and were synthesized from Xcelris Labs Pvt. Ltd, Ahmedabad, India (Table 1). The root samples of healthy banana plants (negative control), banana plants inoculated with CSR-F-1 (Foc TR4), banana plants challenged with CSR-F-1 (Foc TR4) followed by subsequent treatment with the best performing *Trichoderma* isolate (Foc-TR4 + CSR-T-3), and banana plants treated with the best performing *Trichoderma* isolate alone were collected at 30 days after treatment. Total RNA was isolated from the root samples of all four treatments using the Spectrum™ Plant Total RNA Kit (Sigma USA) following the manufacturer's instructions. The quality of total RNA was checked on 1% agarose gel and quantified using a QIAxpert spectrophotometer (Qiagen Germany). The RNA (3 μg) was subjected to cDNA synthesis using a Maxima First Strand cDNA Synthesis Kit. RT-PCR was performed using 10 ng cDNA as the template, forward and reverse primers (5 picomoles each), and the SYBR master mix (ABI, NY, USA) in Applied Biosystems™ 7500 Real-Time PCR Systems (ABI, NY, USA). PCR amplification was programmed with a pre PCR read at 60°C for 30 s, a holding stage at 95°C for 10 min, and a cycling stage at 95°C for 15 s and at 60°C for 1 min. Data were normalized twice, once with banana housekeeping gene encoding disease resistance protein RPM (*MaRPM*) and then with actin genes of *Fusarium* and *Trichoderma* (*FocACT, TrACT*) for assessing the relative abundance of the transcripts related with Fusaric acid biosynthesis and *Trichoderma* related genes, respectively. The differential expression of the genes was expressed in terms of 2-fold change using the formula, Fold Change (FC) = 2^(−ΔΔCt)^.

**Table 1:** Description of primers used for gene expression analysis through RT-PCR.

**Gene**	**Forward primer (5′-3′)**	**Reverse primer (5′-3′)**	**Product size (bp)**
*MaRPM1*	TACACCATTCTCGGCGGCGG	TGCCTCGCCATCACCACCAG	150
*TrACT*	CACCTTCTCCACCACCGCAG	GAGGAGCACGGAATCGGTCG	179
*FocACT*	TCGCCCCCGTCATCATGGTA	GGTTGATGGGAGCCTCGGTG	227
*TrTGA1*	TCCACGAGGGCGGCTACTCT	ACTCCTGCACGCCGTGATCC	249
*TrTMK*	GCCCGATCTGCTGCTTCCCA	AGCCAGGATGCAGCCCACAG	147
*TrVEL*	CGGCACCCTACTCTGCACCC	TGGCGGAGGAGGAAGAGGCT	149
*TrTAC1*	ACCCTCGCTGTTGGAAGCCC	GCTGTTGCGCCAGTTCGACG	127
*TrCBHI*	ACCTTATGGCGAACGGCGAC	TCACAGTACCCCGTGCCGTA	193
*TrCBH2*	AGCAGCCTACCGGACAGCAA	CCGGTTGCAAGGCATCTGGG	204
*TrEGL1*	CCACCACTGGCTCTGGCTCC	AAGTGGCAACAGGAGCGGCA	210
*TrXYN1*	CGAGCCTTCCATCGTCGGCA	TCCTCCCCACCCCTCCACAG	172
*FocFUB3*	GTGCAGAAGCCGTGGGGTCA	ATCGTGCTCGGCCAGGATGG	243
*FocFUB8*	ACAGGTGTCGAGATGCGCCC	CGGGGTGTTTCGGGTGTCGT	157
*FocFUB7*	GGTTCTCTGGCCAGGCTCCG	TGGCATCACCCACGTTGGCA	173
*FocFUB6*	CCAGAGCCCGGCGTCCATAC	GGGGATCGAGACCAGCCGTG	103
*FocFUB9*	TGCCATTGAGCGGGGTGTTGA	GCAATGCGCGTCTTGCCCTT	129

### Biochemical Analysis of Defense-Related Enzymes and Phenol Content

Leaf samples of 1 g (three samples from each replicate) were homogenized with 2 ml of 0.1 M sodium citrate buffer (pH 5.0) at 48°C and centrifuged for 20 min at 10,000 rpm. The supernatant crude enzyme extract was used for assaying 1, 3-glucanase (Pan et al., 1991) and chitinase (Boller and Mauch, 1988) activity. Enzyme extracted in 0.1 M sodium phosphate buffer (pH 7.0) was used for the estimation of peroxidase (PO) (Hammerschmidt et al., 1982), polyphenol oxidase (PPO) (Mayer et al., 1965), and phenylalanine ammonia lyase (PAL) (Ross and Sederoff, 1992). The phenol content was estimated following the procedure of Zieslin and Ben-Zaken (1993) and expressed as catechol equivalents g^−1^ of protein. The assays were carried out for the TC, TF, and TFTR samples and the data were statistically analyzed using the SAS 9.2 statistical software.

### Preparation of Bioformulation of CSR-T-3 and Application Techniques

A 2 mm disc of the best performing *Trichoderma* isolate was inoculated into the CSR patent protected culture media (Rai et al., 2012) and incubated in a rotary shaker at 150 rpm for 72 h at room temperature (28 ± 2°C). After 72 h of incubation, the formulation containing 6 × 10^7^ spores ml^−l^ was used for drenching the plants. The plants were drenched with 500 ml/plant with 1% of the formulation prepared using the best performing *Trichoderma* isolate CSR-T-3 at 2, 4, 6, and 8 months after planting (Damodaran et al., 2019a). The untreated control plants were drenched with the plain culture at the same quantity used in treatment. The incidence of wilt disease in the treatments was scored based on a 1–5 disease scale (Ploetz et al., 1999). Besides disease incidence, observations of growth parameters, such as the girth and height of the pseudo stem, leaf area, yield, and number of hands were recorded according to the guidelines of the International Network for the Improvement of Banana and Plantain (INIBAP).

### Field Evaluation of CSR-T-3 on Disease Affected Hotspot of Uttar Pradesh (India)

A field experiment was performed in the Sohawal block (26° 46′ 3.3852″ N latitude; 81° 59′ 42.7992″ E longitude) of the Faizabad district (Uttar Pradesh, India) during 2018–2019 wherein there was an extensive outbreak reported in 2017 (Damodaran et al., 2018) to evaluate the effectiveness of bioformulation in Fusarium wilt management at field level. The fields (clayey loam soil, pH-7.2) that were reported to have 60–80% *Fusarium* wilt incidence over the past 2 years (2017 and 2018) were selected. Average annual rainfall at the experimentation fields was in the range of 800–900 mm. Disease free banana plantlets of cv. Grand Naine (G-9) produced in the tissue culture laboratory of the ICAR-Central Soil Salinity Research Institute, Regional Research Station, Lucknow, Uttar Pradesh were planted and 50 plants per replication were used for each treatment. The experiment was laid out in a completely randomized block design with two treatments (one treatment included G-9 banana plantlets planted in a Foc infested field while the other treatment included banana G-9 plantlets planted in a Foc infested field and treated with the CSR-T-3 *Trichoderma* isolate formulation). The plants under the treatment were drenched with 500 ml/plant with 3% of the formulation at 2, 4, 6, and 8 months after planting replicated three times with 50 plants in each replication. The fertilizers and farm yard manure (FYM with nitrogen 0.5–1.5, phosphorus 0.4–0.8, and potassium 0.5–1.9) were applied as per the recommended dosage (Crop Production Guide, 2017). The data on growth and yield were recorded and the data on field experiment were subjected to a paired *t*-test (*p* < 0.05) for each parameter and were statistically analyzed using the SAS 9.2 statistical software. The percentage values of the disease index were arcsine transformed (Rangaswamy, 1995).

## Results

### *In vitro* Bioassay of the *Trichoderma* Isolates for Selecting an Elite Antagonistic Isolate Against Foc TR4

**Figure 1:**
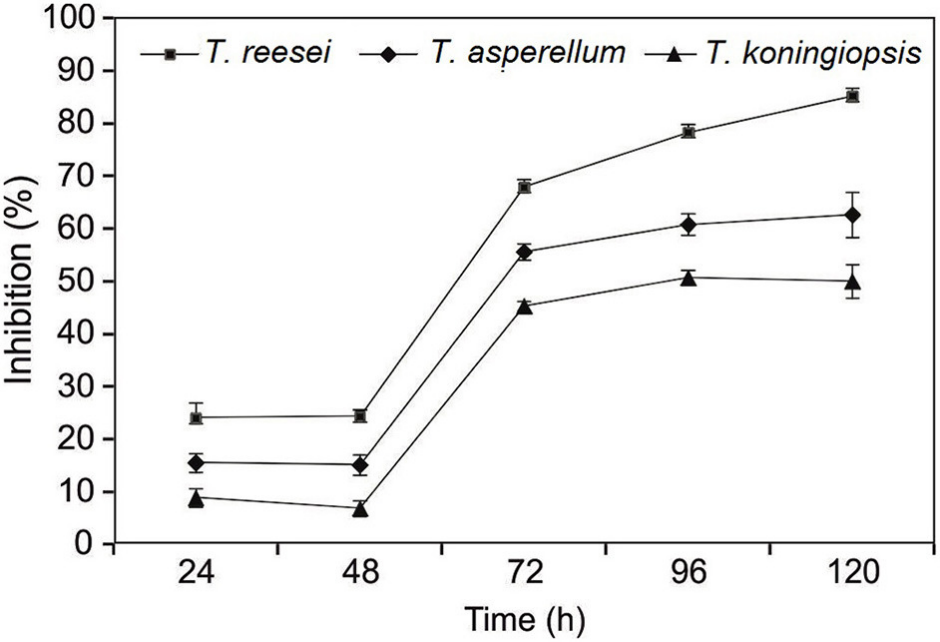
Inhibitory effect of CSR-T-3 (*Trichoderma reesei*) against pathogenic isolate CSR-F-1 of *Foc* TR4. Percentage inhibition of *Foc* TR4 was found to be highest in CSR-T-3 (*Trichoderma reesei*) over a period of time in comparison with *T. asperellum* and *T. koningiopsis*.

Three *Trichoderma* isolates *viz*., *T. reesei* (CSR-T-3), *T. koningiopsis* (CSR-T-2), and *T. asperellum* (CSR-T-4) were assessed for their *in vitro* efficacy by a dual co-culture technique on PDA medium. All of the three isolates utilized the nutrient source and showed a significant increase in the inhibition percentage between 48 and 72 h after inoculation by inhibiting the mycelia growth of Foc TR4 (CSR-F-1). The maximum reduction of the mycelia growth of the pathogen was elicited by the isolate CSR-T-3 that showed the highest inhibition percentage of 85.19% (Figure 1) at 120 h after inoculation, whereas CSR-T-1 showed the least inhibition (50.00%). The inhibition percentage of CSR-T-2 (*T. koningiopsis*) was 62.65%. Based on these results, it is evident that CSR-T-3 (*T. reesei*) is the most effective *Trichoderma* isolate with a tremendous antagonistic ability against Foc TR4.

### SEM Analysis of Dual Culture Interaction to Ascertain CSR-T-3 Antagonism

**Figure 2:**
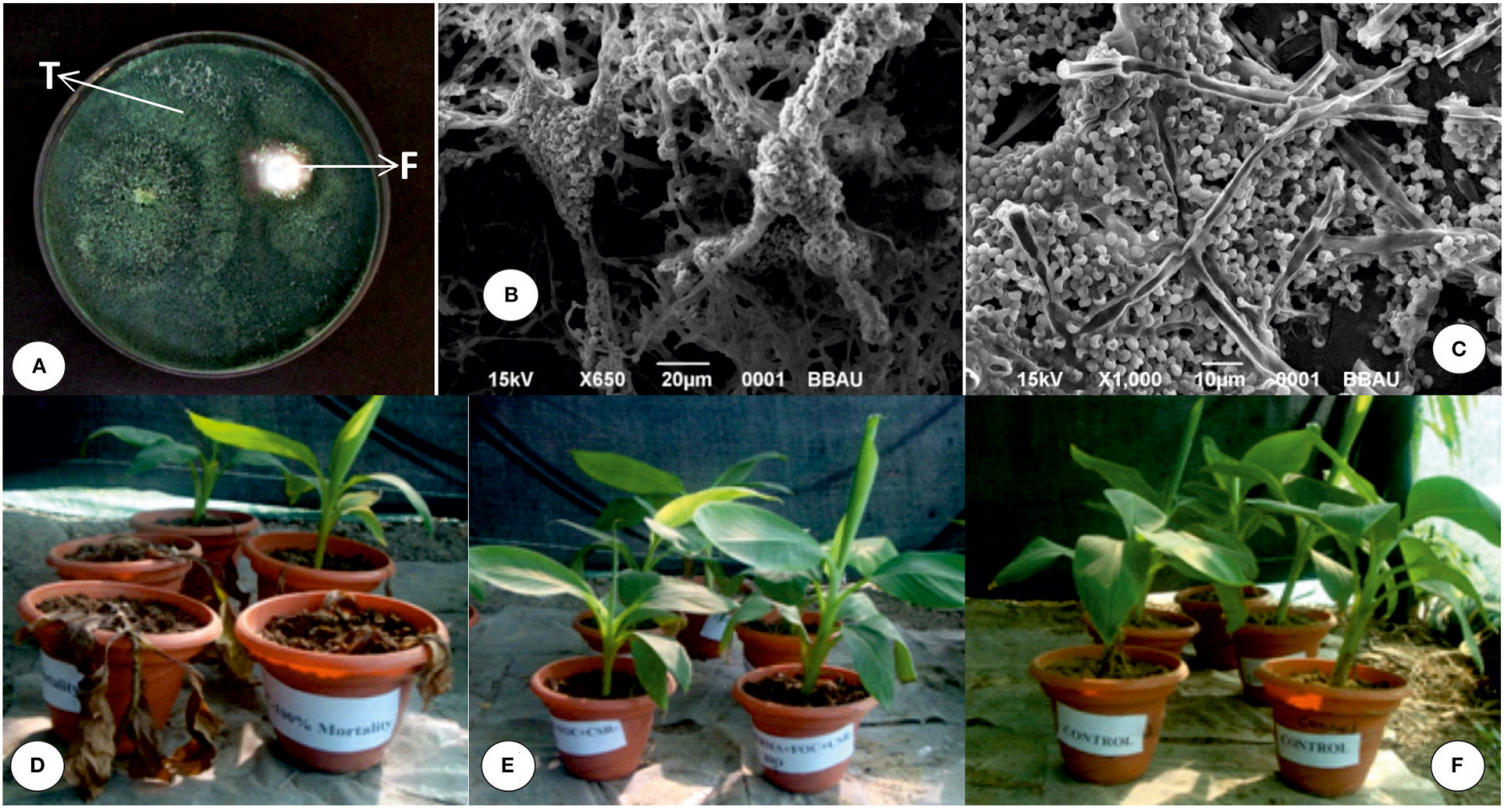
Characterization and evaluation of CSR-T-3 as a biocontrol agent for the management of banana wilt caused by *Fusarium oxysporum* f. sp. *cubense* tropical race 4 (Foc TR4). **(A)** *in vitro* dual culture assay showing inhibition of *Foc* TR4 (F) by the CSR-T-3 *Trichoderma reesei* isolate (T). Scanning electron micrograph showing **(B,C)** mycoparasitism of the *Trichoderma reesei* CSR-T-3 isolate on *Foc* TR-4 mycelium. Pot culture experiment of banana plants for testing the biocontrol potential of the formulation of CSR-T-3 showing **(D)** TF (treatment with *Foc* TR4), **(E)** TFTR (treatment with *Foc* TR4 + CSR-T-3), and **(F)** TC (treatment negative control). Characteristic wilting symptoms manifested in TF **(D)** where the pathogen was challenge-inoculated whereas, the plants treated with CSR-T-3 and challenged with *Foc* TR4 showed healthy growth indicating the protection offered by CSR-T-3 as a biocontrol agent.

SEM analysis of the dual culture interaction zones between the CSR-T-3 (*T. reesei* isolate) and CSR-F-1 Foc TR4 isolates revealed considerable changes in the morphology including collapse, deformation, and broken cells (Figures 2A–C) leading to the loss of integrity of the Foc TR4 hyphae while the spores and mycelium in the control plates were intact and undisturbed leading to normal spore formation. This morphological microscopic identification indicates the mycoparasitism exhibited by CSR-T-3 which is identified as *T. reesei* (NAIMCC-SF-0030; NCBI No. MH997668).

### Green House Evaluation of CSR-T-3 (*Trichoderma reesei*) on *Fusarium* Disease Control

From the *in vitro* antifungal assay of the *Trichoderma* isolates against CSR-F-1 (Foc TR4), *Trichoderma* isolate CSR-T-3 with a significantly high inhibition percentage was used for *in vivo* pot culture studies. The disease spread was rapid and the symptoms of yellowing of older leaves started appearing in the Foc TR4 inoculated plants from 24 days after planting in control plants (Figure 2D; additional root morphological differences in Supplementary Figure 3). As time went on the plants in the Foc TR4 alone treatment began to show mortality between 60 and 90 days. At 90 days after planting when the experiment was completed, the plants that had been treated with Foc TR4 showed a significantly high mean disease severity index of 3.75 while the plants treated with TFTR recorded significantly lower DSI (0.75) that was similar to the plants of control that were not inoculated with the pathogen (Table 2). None of the plants treated with TFTR showed mortality until the end of the experiment (Figure 2E).

**Table 2:** Effect of *Trichoderma reesei* (CSR-T-3) on *Fusarium* wilt incidence and growth of banana plantlets under greenhouse pot experiment.

**Treatment**	**Plant height**			**Plant girth**			**No. of leaves**	**Disease severity index**
	**Initial**	**Final**	**Increment**	**Initial**	**Final**	**Increment**		
TF	17.15^b^ (±1.63)	26.5^a^ (±4.40)	33.83^a^ (±13.17)	3.35^a^ (±0.17)	3.62^a^ (±0.17)	7.51^a^ (±4.51)	1.75^a^ (±1.25)	3.75^b^ (±1.50)
TFTR	14.9^b^ (±2.01)	33.75^b^ (±1.49)	55.79^b^ (±6.29)	3.50^a^ (±0.40)	5.47^b^ (±0.55)	35.82^b^ (±7.44)	4.50^b^ (±0.57)	0.75^a^ (±0.50)
TC	13.12^a^ (±1.51)	35.47^b^ (±1.94)	62.89^b^ (±5.14)	3.80^a^ (±0.35)	5.55^b^ (± 0.17)	31.63^b^ (±4.35)	5.00^b^ (±0.00)	0.00^a^ (±0.00)

*TF, treatment with Fusarium; TFTR, treatment with Fusarium and Trichoderma; TC, treatment control*.

*Values are the means of five replicates with the sample size n = 5*.

*Means in the columns followed by the same letter (either a or b) are not significantly different according to Duncan's multiple range test at P = 0.05. Values in the parentheses indicate the standard deviation of the mean*.

Plant height and girth in the TFTR-treated plants were recorded to be 33.75 and 5.47 cm, respectively which were significantly higher than the TF-treated plants (Foc TR4 alone treated banana plants). Interestingly, there were no significant difference in height and girth observed in the TFTR-treated plants (*T. reesei* isolate CSR-T-3 treated banana plants with challenge inoculation of Foc TR4 strain) as well as in the case of TC (untreated negative control). About a 55.79% increase in plant height was noticed in plants with TFTR treatment which also exhibited increased stem girth by 35.82% in comparison with plants with Foc TR4 alone treatment (TF) (Figures 2E,F). The results suggest that the treatment of the fungi *T. reesei* (CSR-T-3) significantly reduced the incidence of the disease and also promoted the growth of the seedlings on par with that of healthy plants.

### Effect of *Trichoderma reesei* CSR-T-3 on Biomass and Chlorophyll Content of Bananas

**Figure 3:**
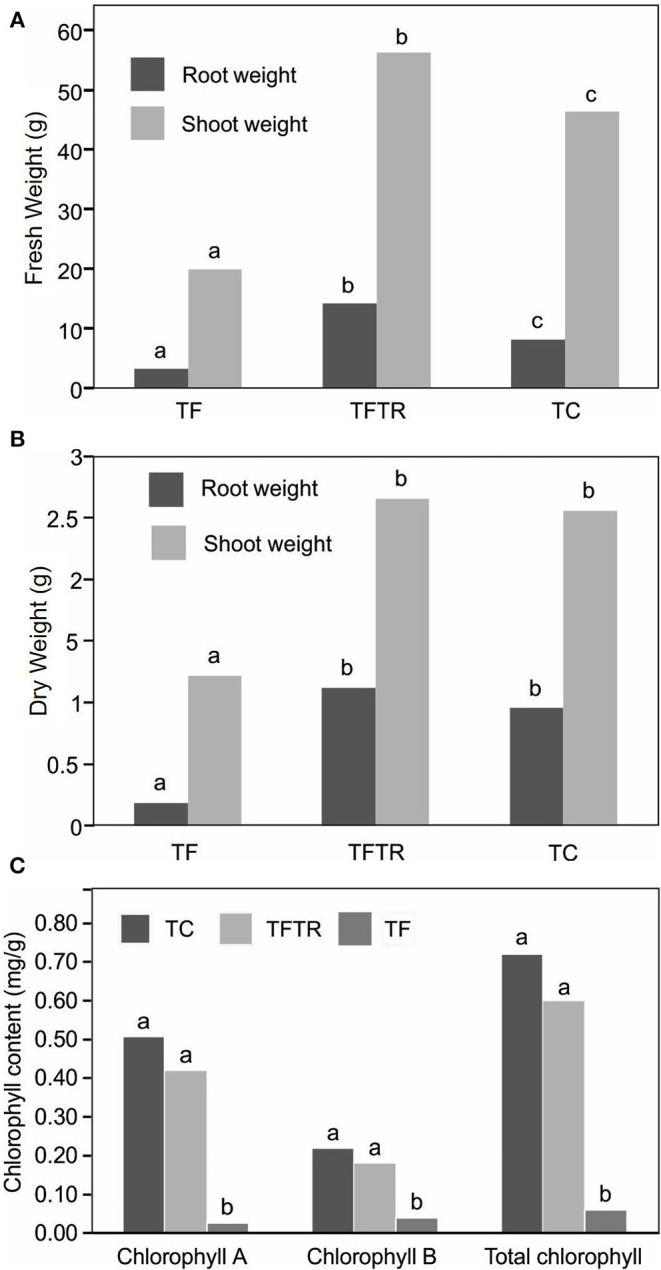
Effect of CSR-T-3 (*Trichoderma reesei*) on banana root and shoot tissues, **(A)** fresh weight in “g” and **(B)** dry weight in “g” under greenhouse pot experiment while **(C)** shows variations in chlorophyll content. Both fresh and dry weights of shoot and root tissues were found to be enhanced in TFTR indicating growth promotion aided by the treatment in comparison with control. Quite contrarily, the TF plants showed reduced shoot and root weights (fresh and dry) which could be attributed to the wilting and senescence of the tissues. Chlorophyll a and b as well as total chlorophyll content reduced in TFTR and TF over the control TC.

Although, there was no improvement in plant height and pseudo stem girth in CSR-T-3 treatment, significant differences were recorded for growth parameters like weight of the pseudo stem and root. The mean fresh (Figure 3A) and dry (Figure 3B) weight of the plantlets treated with the *T. reseei* CSR-T-3 isolate were higher compared to those in the other treatments including control without any microbial inoculations (CSR-T-3, antagonistic biocontrol agent and CSR-F-1, Foc TR4 pathogen). The fresh root and shoot weight for the TFTR treatment (14.20 and 56.30 g, respectively) inoculated with CSR-T-3 *T. reesei* isolate along with Foc TR4 was significantly higher than the TC control and TF treatment. However, there was no significant difference between root and shoot dry weights in the TFTR treatment (1.11 and 2.60 g, respectively) and TC which were significantly higher than the TF treatment (0.96 and 2.60, respectively). Thus, treatment with the *T. reesei* CSR-T-3 isolate had a growth promoting effect on banana plants. Similarly, total chlorophyll content 0.60 mg/g was recorded in the TFTR treatment, which was significantly higher than the TF treatment (value). The total chlorophyll content in the untreated control TC treatment (0.72 mg/g) and TFTR treatment showed non-significant differences indicating the effect of the treatment in reducing the stress of the plants due to the inoculation of the pathogen (Figure 3C). Overall, it was observed that there was additional growth improvement noticed in the CSR-T-3 treatment apart from disease suppression, despite the stress imposed by the inoculation of Foc TR4. The growth attributes were found to be on par with the healthy plants.

### Metabolite Profiling of CSR-T-3 Efficacy Using LC-MS Analysis

**Figure 4:**
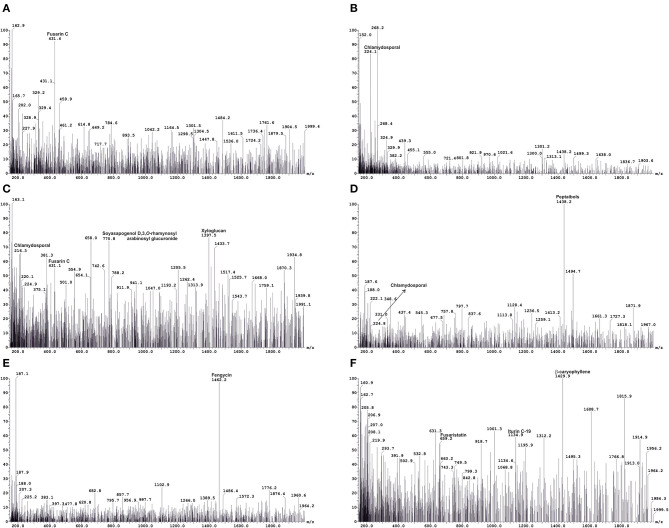
A representation of liquid chromatography-mass spectrometry (LC-MS) analysis used for identification of key metabolites produced by host-pathogen interactions. Metabolite profiling using LC-MS indicating the production of pathogen-related toxins, such as fusarin C and chlamydosporal **(A,B)** in *Foc* TR4-infected wilted plants (TF) and detection of antifungal compounds **(C–F)**, such as ß caryophyllene, soyasapogenol D, 3-*O*-rhamnosyl arabinosyl glucuronide, peptaibols, and fengycinin CSR-T-3 treated banana plants challenged with *Foc* TR4 (TFTR). Simultaneous detection of pathogen-related toxins at low intensity **(C,D)** indicating a reduction of toxins and increase in antifungal metabolites under CSR-T-3 TFTR treatment.

The crude metabolites of plants treated with Foc TR4 (TF), Foc TR4, and CSR-T-3 (TFTR), and untreated negative control (TC) were analyzed by LC-MS to detect the production of antimicrobial compounds by CSR-T-3 as well as Fusarium toxins. LC-MS spectral profiles with different retention times and relative intensity (%) facilitated the identification of the compound showing distinct peak values by comparing their identity using similar molecular mass (m/z) values reported for distinct compounds (Figure 4). The compounds identified from LC-MS analysis could be broadly categorized as phenolic esters, antioxidants, fatty acids, fungal toxins, and compounds with antifungal and antibacterial principles. The extracts from the treatment control (TC) showed distinct peaks for fatty acid compound 20-methyl spirolide G (MeG) and soyasapogenol e-3-*o*-rhamnosyl glucosyl glucuronide (Table 3). The compounds in the crude extract of TFTR treatment with relative abundance was identified as ß-caryophyllene (1,429.9), catechin-o-gallate, soyasapogenol rhamnosyl glucoronide (729), peptaibols (1,788.2), fenigycin (1,462.2), iturin C19 (1,134.9), anthocyanin (1,191.7), and gallocatechin-o-gallate (913.5) (Figures 4C–F). Fungal toxins and metabolites produced by *Fusarium* spp. like enniatin A, fusarin C, chlamydosporal, etc. were also identified in TFTR treatment with low peak intensity (Figures 4C,D). The extracts of treatment Foc TR4 showed distinct compounds like fusaristatin A, fusarin C, chlamydosporal, and beauveric acid with high peak intensity (Figures 4A,B).

**Table 3:** Key metabolites identified from LC-MS analysis of crude extracts of the banana root samples under various treatments.

**Treatments**	**Compound**	**Peak value**	**RT (min)^**a**^**	**Intensity (%)**	**Activity**	**References**
^*^TC	Methyl esters of 20-methyl spirolide G (MeG)	887.6	24.96	100	Fatty acid	Aasen et al., 2006
	6″-p-coumaroylglucoside	609.0	9.21	100		Trikas et al., 2016
	Soyasapogenol E-3-*O*-rhamnosyl glucosyl glucuronide	793.5	26.64	100	Antifungal, antibacterial	Sagratini et al., 2009; Pollier et al., 2011
^**^TFTR	Catechin-*o*-gallate	729	29.03	44	Antioxidant	Tala et al., 2013
	ß caryophyllene	1429.9	2.22	100	Antifungal	Qi et al., 2017
	Enniatin A	687.3	21.56	25	Secondary metabolite *Fusarium* sp.	Sorensen and Giese, 2013
	Soyasapogenol E-3-*O*-rhamnosyl glucosyl glucuronide	793.6	21.56	100	Antifungal, antibacterial	Sagratini et al., 2009; Pollier et al., 2011
	Peptaibols	1438.2	7.13	100	Antifungal	Mukherjee and Kenerley, 2010
	Xyloglucan	1397.5	5.08	80	Antifungal	Vinueza et al., 2013
	Soyasapogenol D-3-*O*-rhamnosyl Arabinosyl glucuronide	770.8	5.08	75	Antifungal	Jin et al., 2006, 2007; Tava et al., 2009, 2011
	Fenigycin	1462.2	7.40	100	Antifungal	Pathak and Keharia, 2014
	Iturin C19	1134.9	2.22	60	Antifungal	Pathak and Keharia, 2014
	Anthocyanin	1191.7	7.98	70	Antioxidant	Hwang et al., 2017
	(Epi)gallocatechin-O-gallate	913.5	13.58	100	Antioxidant	Tala et al., 2013
^***^TF	1-Palmitoyl-2-10-hydroxy-5,8,11-tridecatrienoic acid	732.6	17.25	100	Host defense	Reis et al., 2005
	Gallocatechin-O-gallate	913.5	13.61	100	Antioxidant	Tala et al., 2013
	Beauveric acid	801.7	27.33	25	Fungal toxin	Li et al., 2013
	Fusaristatin A	659.8	1.81	20	Fungal toxin	Hegge et al., 2015
	Fusarin C	431.4	10.27	100	Fungal toxin	Sorensen and Giese, 2013
	Chlamydosporal	224.1	1.48	85	Secondary metabolite of *Fusarium* sp.	Sorensen and Giese, 2013

* TC, treatment control;

** TFTR, treatment with Foc TR4 and Trichoderma reesei isolate CSR-T-3;

*** *TF, treatment with Foc TR4 alone*.

### Gene Expression Profiling of Banana Plants With CSR-T-3 Treatment

**Figure 5:**
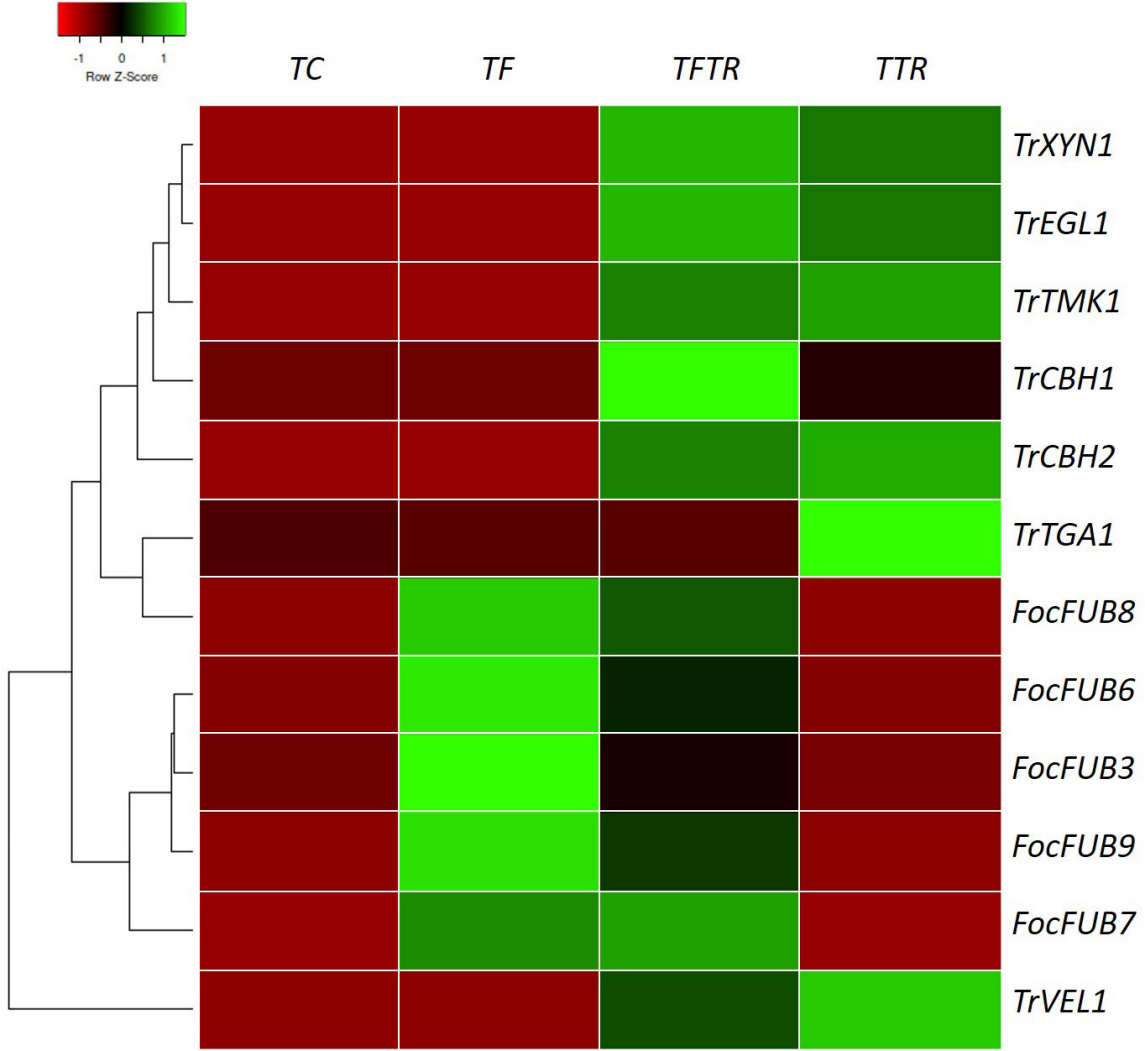
Heat map showing gene expression patterns of key genes involved in fusaric acid biosynthesis by *Foc* TR4, mycoparasitism, and secondary metabolite production inducing signal transduction pathway genes of *Trichoderma reesei* in various treatments for establishing the biocontrol mechanism of CSR-T-3. Fusaric acid biosynthesis involves a series of genes, such as *FUB3, FUB6, FUB7, FUB8*, and *FUB9*. Fusaric acid is one of the major toxins produced by *Fusarium oxysporum* f. sp. *cubense* tropical race 4 and the upregulation of these genes in banana plants challenge-inoculated with Foc TR4 (TF) while downregulation of the same was recorded in TFTR (CSR-T-3-treated banana plants challenge-inoculated with *Foc* TR4) and TTR (CSR-T-3-treated banana plants without pathogen). Similarly, the genes involved in mycoparasitism (*TrCBH1, TrCBH2, TrEGL1, TrXYN1*) and those involved in the signal transduction pathway inducing secondary metabolite production (*TrVEL1, TrTGA1, TrTMK1*) were found to be significantly upregulated in TTR and TFTR. Green color code represents upregulation and red color code indicates downregulation. TC indicates negative control (no pathogen, no biocontrol), TF represents *Foc* TR4-challenged banana plants, TFTR indicates CSR-T-3-treated banana plants challenged with *Foc* TR4 and TTR indicates positive control (banana plants with CSR-T-3 (*Trichoderma reesei* isolate).

Gene expression profiling revealed that the expression levels of *Fusarium*-related genes indicated a clear pattern of upregulation of the Fusaric acid biosynthesis genes in only *Fusarium* treatments (TF and TFTR) evident from the heat map presented in Figure 5. In the case of *Trichoderma* mycoparasitism genes (*TrCBH1, TrCBH2, TrEGL1, TrXYN1*) and signal transduction pathway genes for secondary metabolite production (*TrTGA1, TrTMK1*, and *TrVEL1*) a distinct pattern of upregulation was recorded in only the *Trichoderma* treatments (TFTR and TTR) (Figure 5). The significant upregulation of the Fusaric acid biosynthetic genes in TF and their corresponding downregulation in TFTR confirmed the suppression of Fusarium toxin production by *T*. *reesei*. An exception was recorded in the expression levels of the *FUB3* gene which showed a trend toward no change in TFTR (Z score-0). Contrarily, the significant downregulation in TTR (i.e., almost no expression) also strongly affirms the absence of the pathogen Foc TR4 and thereby toxin pathway gene expressions are being curtailed. On the other hand, the mycoparasitism-related genes, such as *TrCBH1, TrCBH2, TrEGL1, TrXYN1* were found to be significantly upregulated in TFTR and these genes were involved in the breakdown of the Foc cell wall and their components and are being produced by CSR-T-3. Interestingly, the *TrCBH1* gene seems to be an inducible gene upon pathogen invasion as seen in the change from Z-score 0 (no change) in TTR toward +1 (significant upregulation) in TFTR. But other genes seemed to exhibit constitutive expression as both TFTR and TTR showed similar levels of upregulation. Some signal transduction pathway genes, such as *TrVEL1* and *TrTMK1* were found to be significantly upregulated in TFTR which was similar to the pattern exhibited by TTR even though an upstream gene *TrTGA1* showed downregulation in TFTR which was observed to be upregulated in TTR.

### Possible Biocontrol Mechanism Elucidated From Gene Expression and LC-MS Analysis

**Figure 6:**
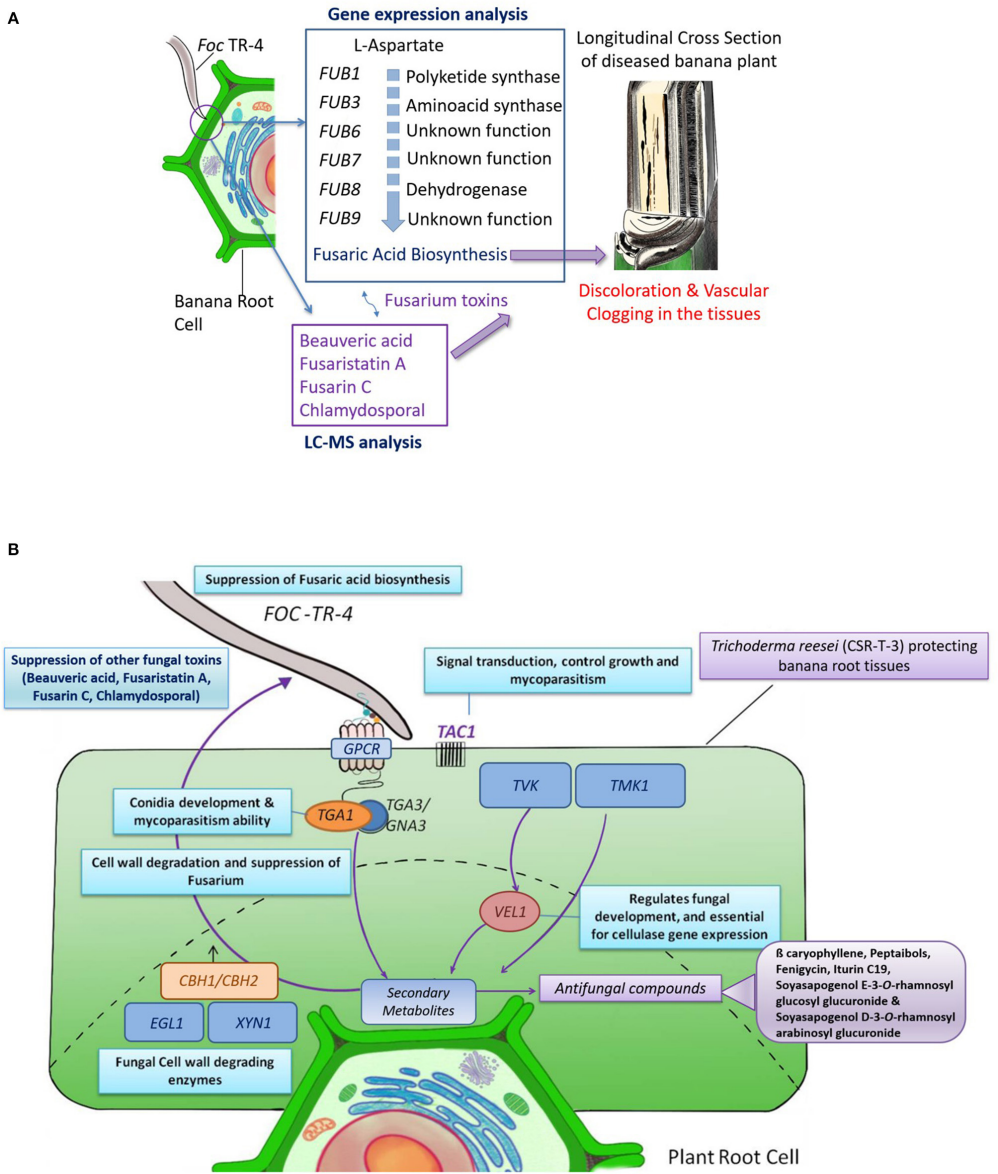
Possible mechanism of CSR-T-3 (*Trichoderma reesei*) in providing protection against Foc TR4 pathogen in bananas. **(A)** Mode of infection in the banana plants are induced by the toxins produced by *Fusarium oxysporum* f. sp. *cubense* tropical race 4 (*Foc* TR4) which was evident from the gene expression analysis and LC-MS profiling. The toxins and the mycelial mats results in discoloration and vascular clogging of the plant tissues, the most characteristic symptom of *Foc* TR4. **(B)** Mechanism of CSR-T-3 through the expression of mycoparasitism-related genes cellobiohydrolase (*CBH1*/*CBH2*), endoglucanse (*EGL1*), and xylanase (*XYL1*) genes and the signal pathway that are induced by *TMK1* and *VEL1* genes are evident from the gene expression studies. Expression of these genes also triggers the production of secondary metabolites especially antifungal compounds, such as ß-caryophyllene, catechin-o-gallate, soya sapogenol rhamnosyl glucoronide, peptaibols, fenigycin, iturin C19, anthocyanin, and gallocatechin-o-gallate. Co-suppression of fusaric acid and other toxins, such as beauveric acid, fusarastatin A, fusarin C, and chlamydosporal during the same time results in conferring tolerance in the banana plants.

LC-MS and gene expression studies have provided some insights on the probable mode of action which is proposed as a probable mechanism of biocontrol exhibited by the CSR-T-3 *Trichoderma reesei* isolate against Foc TR4 (Figure 6). The role of Fusaric acid was confirmed from gene expression analysis but was not evident from LC-MS analysis. Fusaric acid is the major toxin reported in many Fusarium species. Fusaric acid biosynthesis involves several enzymes polyketide synthase (*FUB1*), aminoacid kinase (*FUB3*), hydrolase (*FUB4*), acetyl transferase (*FUB5*), dehydrogenase (*FUB8*), and proteins of unknown function (*FUB2, FUB6s*, and *FUB9*). But in the present study, from the LC-MS analysis, the role of other toxins, such as fusaristatin A, fusarin C, chlamydosporal, and beauveric acid could be established to be present in infected plants. Based on the time and location of the sampling for LC-MS analysis, it is evident that Fusarium-related toxins are accumulated in the Foc TR4-infected plants which are responsible for the discoloration (Figure 6A). The mycelial growth of the pathogen and establishment in the plant tissues caused vascular clogging thereby blocking nutrient-water movement and eventually resulting in the sudden wilting of the plants. The counteracting mechanism of biocontrol agent CSR-T-3 by mycoparasitism and production of antifungal metabolites was established by the results observed in gene expression and LC-MS analysis. The typical signal transduction pathway, especially the genes upstream to the production of secondary metabolites induction, as well as those associated with the antifungal properties were upregulated in both TFTR and TTR. This was supported by the downregulation of Fusarium-related toxins and the upregulation of antifungal compounds in the LC-MS data. Thus, a possible mechanism of biocontrol potential of the CSR-T-3 *T. reesei* isolate against Foc TR4 has mycoparasitism and antifungal metabolites as key contributors (Figure 6B).

### Biochemical Analysis of Defense-Related Enzymes and Phenol Content

Stress conditions are known to induce reactive oxygen species (ROS) production thereby resulting in oxidative damage in the cells. This effect can be recorded by assaying and quantifying the ROS scavenging enzymes which may be produced by the host banana plants upon infection with Foc TR4. Sometimes, the biocontrol agents also facilitate production and induction of host defense enzymes. Assaying the defense enzymes in different treatments was completed with the aim of assessing the host response in combination with the CSR-T-3 treatment (Table 4). The accumulation of defense enzymes and phenols was significantly higher in the TFTR treatment (where plants were treated with the pathogenic isolate of Foc TR4 and the *T. reesei* CSR-T-3 isolate) in comparison with TF (Foc TR4-treated) and TC (negative control with no treatments). The treatments involving *T. reesei* isolate CSR-T-3 invariably had a higher induction of peroxidase (POD), polyphenol oxidase (PPO), phenylalanine ammonia lyase (PAL), and ß-1, 3-glucanase activity than the TF and TC treatments. Significant induction of chitinase activity (3.160-fold increase in nmol of GlcNAc g^−1^fw min^−1^) was observed in *T. reesei* isolate CSR-T-3 inoculated TFTR treatment while Foc TR4 alone inoculated TF treatment recorded induction but at low levels. The treatments involving *T. reesei* isolate CSR-T-3 had nearly 1.382 times higher phenolic content than the control plants.

**Table 4:** Profiling changes in activities of defense enzymes in *Trichoderma reesei* (CSR-T-3)-inoculated banana plantlets challenged with pathogenic isolate CSR-F-1 of Foc TR-4.

**Treatment**	**Peroxidase (ng^**−1**^fw min^**−1**^)**	PPO* (ng^**−1**^fw min^**−1**^)	PAL** (nmol of trans-cinnamic acid g^**−1**^fw min^**−1**^)	**β-1, 3-glucanase (mg of glucose g^**−1**^fw min^**−1**^)**	**Chitinase (nmol of GlcNAc g^**−1**^fw min^**−1**^)**	**Phenols (μg/g fresh weight)**
TC	19.463^a^ (±0.61)	0.136^a^ (±0.61)	1386.570^a^ (±42.23)	198.800^a^ (±11.14)	25.560^a^ (±8.57)	378.400^a^ (±25.11)
TF	20.154^a^ (±0.57)	0.176^a^ (±0.01)	2020.630^b^ (±59.71)	225.600^b^ (±14.14)	33.290^b^ (±2.22)	463.600^b^ (±16.15)
TFTR	25.526^b^ (±0.74)	0.187^a^ (±0.01)	2688.030^c^ (±81.33)	316.560^c^ (±5.15)	80.778^c^ (±3.16)	523.000^c^ (±26.02)

TF, treatment with Fusarium; TFTR, treatment with Fusarium and Trichoderma; TC, treatment control; PPO^*^, Polyphenol oxidase; PAL^*^, Phenylalanine ammonia lyase.

*Values are the means of five replicates with the sample size n = 5*.

*Means in the columns followed by the same letter (a, b, or c) are not significantly different according to Duncan's multiple range test at P = 0.05. Values in the parentheses indicate the standard deviation of the mean*.

### Development of Bioformulation and Field Evaluation of CSR-T-3 (*T. reesei*) Bioformulation

Bioformulation developed using the *T. reesei* CSR-T-3 isolate that showed promising results in pot culture studies under greenhouse conditions (Figure 2) in terms of disease suppression was taken to the field level evaluation in the hotspot region of the wilt disease. Efficacy of CSR-T-3 formulation was tested on sick plots in the hot spot area of Sohawal (26° 46′ 3.3852″ N latitude; 81° 59′ 42.7992″ E longitude) where there were severe disease incidences during 2017 and 2018 (Damodaran et al., 2019c). The CSR-T-3 *T. reesei* isolate was formulated in the form of bioformulation using a loopful of a 2 mm disc in the CSR patent protected bio-media and incubated for 72 h. After 72 h, the plants under the treatment were drenched with 500 ml/plant with 3% of the formulation at 2, 4, 6, and 8 months after planting.

Typical wilting symptoms of yellowing in the older leaves started in the untreated control plants from the 4th month (November) after planting followed by pseudo stem splitting at the 6th month (January) after planting with the disease spreading rapidly over 12–14 months after planting (July-August) corresponding with the flowering and fruiting stages. High disease incidence percentage (46.50%) (Table 5) was recorded in the plants that were not treated with CSR-T-3 *T. reesei*. However, there was a significant reduction in the disease incidence percentage (10.58%) of the plants treated with *Trichoderma* isolate CSR-T-3. At harvest, the disease severity index of the *Trichoderma-*treated plants were significantly lower (1.14) compared with those that were not treated (3.46). The plant height (230.73 cm), girth (43.9 cm), and leaf area (73.13 cm^2^) of the treated plant group were significantly higher than the other untreated control group. The mean yield (30.57 kg/plant) of the treated plants were significantly higher compared to the untreated plants (7.71 kg). However, there was no significant difference in the number of leaves and number of hands between the treated and untreated plants. There was a significant increase in bunch size and finger size that was attributed to enhanced yield over the diseased plant. The yield under treatment was found to be on par with the average yield of the locality that ranged from 25 to 30 kg/plant. Thus, the drenching of *T. reesei* isolate CSR-T-3 not only had an antifungal effect in the control of the disease but also had a growth promoting effect in increasing certain growth parameters, such as plant vigor, finger, and bunch size besides yield (bunch weight) which increased 2-fold over untreated control plants.

**Table 5:** Effect of *Trichoderma reesei* (CSR-T-3) on *Fusarium* wilt and the growth of banana plantlets under field experiment in sick fields.

**Treatment**	**Plant height (cm)**	**Girth (cm)**	**No. of leaves**	**Leaf area (cm^**2**^)**	**Yield (kg/plant)**	**No. of hands**	**Disease incidence (%)**	**Disease severity index**
CSR-T-3	230.73^a^ (±4.70)	43.9^a^ (±7.33)	7.86^a^ (±0.38)	73.13^a^ (±7.14)	30.57^a^ (±3.60)	8.14^a^ (±0.69)	10.58^a^ (±4.64)	1.14^a^ (±0.69)
Control	214.76^b^ (±2.73)	36.1^a^ (±9.72)	6.71^b^ (±0.76)	51.84^b^ (±6.40)	16.29^b^ (±6.13)	7.71^a^ (±1.11)	46.50^b^ (±18.33)	3.46^b^ (±0.51)

*Values are the means of ten replicates*.

*Means in the columns followed by the same letter are not significantly different according to paired t-test at P = 0.05. Values in the parentheses indicate the standard deviation of the mean*.

## Discussion

The global banana production system has been exposed to serious threats in the mid-twentieth century by the devastating wilt disease caused by *F. oxysporum* f. sp. *cubense* (Stover, 1962). Popularly known as Panama disease it wiped out the Gros Micheal cultivation in Central America (Ploetz, 1992). The banana industry was sustained with the identification of the Cavendish (AAA) cultivar as a resistant source for the Foc race 1 pathogen. However, the choice varieties of the Indian sub-continent comprising of Pisang Awak (ABB), Rasthali/Amritpani (AAB), and Malbhog (AAB) still remained highly susceptible to the Foc race 1 and are threatened with extinction (Thangavelu et al., 2001). In the early 1990s, a new race was identified in the tropics, in a much more virulent form, devastating the Cavendish cultivars and designated as tropical race 4 (TR4) that causes 70–100% loss (Ploetz and Churchill, 2011) and also economic loss amounting to US$ 388.1 million (FAO, 2017). The perennial nature of the pathosystem complicated the development of sustainable control measures (Ploetz, 2007). The chance of obtaining a tolerant variety was very difficult due to the existence of extremely poor fertility in the Cavendish cultivars (Ortiz, 2013). However, the utilization of antagonistic microorganisms from suppressive soils, such as *Pseudomonas, Bacillus*, and *Trichoderma* species can aid in the curtailment of the growth and development of the pathogen (Deng et al., 2013). Even among the antagonistic microbes, *Trichoderma* has been widely exploited as the potential biocontrol agent owing to their distinct properties in the control of soil borne disease and non-eco-toxic nature of the genus (Benitez et al., 2004). The fast growing and mycoparasitism ability of the *Trichoderma* isolates place them as potential antagonistic microbes (Monfil and Casas-Flores, 2014; Sellamani et al., 2016). Prominent inhibitory effects of different *Trichoderma* isolates against Fusarium diseases by mycotoxin production were established and indicated the further need of their ability through field validation (Tian et al., 2018).

Success of biological control in the field depends on the correct identification of potential strains and application in the field (Chaves et al., 2011). Therefore, isolation and screening of the highly efficient microorganisms form the base of biocontrol. An attempt was made to assess the efficacy of three *Trichoderma* isolates that were isolated from the rhizosphere of biotic (banana wilt suppressive) and abiotic (banana salt stressed) stress eco-system (Damodaran and Mishra, 2016; Damodaran et al., 2019c). These antagonists exert differential inhibition potential to the pathogen growth that was evident from the *in vitro* assay and the dominating role of disease suppression with isolate CSR-T-3 was successfully demonstrated in the *in vivo* pot culture study. PGPR strains, such as *Pseudomonas fluorescens* PF-1 and *Trichoderma* species TRC-54 were earlier reported to have controlled the mycelia growth of Foc race 1 of banana under a dual culture study through the direct action of enzymes and metabolites produced by PGPR (O'Sullivan et al., 1992; Nagarajkumar et al., 2004). CSR-T-3 was the first strain of *Trichoderma reesei* isolated from the salt affected rhizosphere to show disease suppression against the banana Foc TR4. The antifungal property was further demonstrated in this study using a pot and field experiment. Application of the isolate successfully reduced the disease severity index significantly when compared to Foc TR4-infected plants and also promoted the growth of the banana crop successfully. Earlier, there were successful reports on the biological control of Fusarium wilt disease in bananas using bacterial strains like bacterial isolate ITBB B5 1 (Tan et al., 2015), *Streptomyces* strain CB75 (Chen et al., 2018), and *Bacillus flexus* strain Typr1 (Thangavelu and Gopi, 2015) under pot experiments. But none of these reports have indicated field level application and validation of these strains for commercial use. We report here the first successful report of a fungal strain CSR-T-3 controlling Foc TR4 causing wilt disease in bananas at a field level besides pot culture studies.

*Trichoderma* spp. are known to produce a wide range of bioactive secondary metabolites that are known to have antifungal, antibacterial, and toxic properties to control a wide range of phytopathogens, such as *Alternaria alternata, Botrytis cinerea, Fusarium* spp., *Pythium* spp., *Rhizoctonia solani, Sclerotinia sclerotiorum*, and *Ustilago maydis* (Hyder et al., 2017; Sood et al., 2020). *Trichoderma* spp. is reported as an inexhaustible source of antibiotic derivatives (from the acetaldehydes to alpha-pyrones) terpenes, polyketides, isocyanide derivatives, piperacines, and complex families of peptaibols (Keszler et al., 2000; Sood et al., 2020). Therefore, in order to characterize and profile the metabolites, LCMS analysis was performed which aided in the identification of microbial metabolites who were produced by both the pathogen (CSR-F-1) and the biocontrol agent (CSR-T-3). The treatment involving Foc TR4 challenge inoculation and *T. reesei* isolate CSRT-3 showed the production of a wide range of bioactive antifungal and antioxidant metabolites with high intensity m/z peaks. They include peptaibols, fenigycin, xyloglucan, soyasapanogenol rhamnosyl glucuronide, iturin C, ß-caryophyllene, and antioxidants like catechin-O-galate. The presence of *Fusarium* related metabolites like fusaristatin, fusarin C, chlamydosporal, enniatin C, etc. were also observed but the intensity of the m/z peaks were comparatively lower than the antifungal and antioxidant metabolites in this treatment. However, significant production of *Fusarium*-related metabolites like Fusarin C with a high intensity m/z peak was observed in the TF treatment where only the Foc TR4 isolate was inoculated. This implies that there is a suppression of toxins produced by Foc TR4 in the presence of the antifungal metabolites produced by *T. reesei* isolate CSR-T-3 in this present study. Peptaibols are small peptides of α-aminoisobutyric acid (Aib) produced by the genus *Trichoderma* that are economically important for their anti-microbial and anti-cancer properties as well as their ability to induce systemic resistance in plants against microbial invasion (Mukherjee et al., 2013). The involvement of these compounds in mycoparasitism established with their role in induced systemic resistance in plants by both apoptosis and autophagy suggest their capability in preventing the invasion of root-invading fungi (Schuster and Schmoll, 2010). The intensity of mass peaks assigned for iturin C and fengycin in the TFTR treatment in our study explicitly show the high antifungal potency of the *T. reseei* CSR-T-3 isolate. The cyclic heptapeptide iturin with ß-amino fatty acid was known to have high hemolytic and antifungal properties while fengycin, a cyclic depsipeptide with 10 amino acids and a ß-hydroxy fatty acid tail, is known for its excellent antifungal potential against filamentous fungi (Winkelmann et al., 1983; Vanittanakom et al., 1986). Pathak and Keharia (2014), reported iturin C and fengycin as an antifungal compound produced by *Bacillus subtilis*. Phenolic compounds have crucial roles in inducing the plant defense mechanism through free radical termination by providing hydrogen to reduce free radicals. The presence of phenolic compounds, such as gallocatechin-o-gallate in our LCMS fractions in TFTR and TF treatment signifies the activities of plant defense systems when exposed to biotic stress. The bacterization of tomato plants with beneficial strains showed antifungal activity through the deposition of phenolic compounds and restricting the growth of *F. oxysporum* f. sp. *lycopersici* (M'Piga et al., 1997).

Our study has demonstrated the possibility of the increase in the plant defense system through an increase in the activities of phenols and enzymes like peroxidase (POD) and phenylalanine ammonia lyase (PAL). Peroxidase is a defense enzyme implicated in the last enzymatic step of lignin biosynthesis that oxidizes the hydroxyl cinnamyl alcohols into free radicals that are later coupled with lignin polymer (Gross, 1980). POD is known to inhibit the spore germination and mycelia growth of some pathogenic fungi like *Pseudocercospora abelmoschi* and *Pseudocercospora cruenta* (Joseph et al., 1998). Enhanced POD and PAL activities were reported by Kavino et al. (2007) in bacterial strain-treated banana plantlets that showed tolerance to *Banana bunchy top virus*. PAL is an enzyme that plays an important role in the biosynthesis of defense chemicals in phenyl propanoid metabolism (Daayf et al., 1997). Another prominent group of enzymes and compounds that are commonly correlated with the tolerance mechanism exhibited by the *Trichoderma* spp. are ß-glucanase and chitinase (Chet, 1987). ß-glucans are polysaccharides that stimulate the host immune response on interaction (Garfoot et al., 2014). The higher activity of ß-glucanase and chitinase in the *T. reesei*-treated plants was observed in biochemical assays of our study which could be another additional mechanism attributing to increasing the host immune response to the pathogen. Radjacommare et al. (2004) assessed the chitinoyltic enzyme against *Rhizoctonia solani* in rhizobacteria-treated rice which induced a defense response in the host by bacterial co-inoculants of *Pseudomonas* sp. Higher activity of ß-glucanase and chitinase was present in *F. oxysporum* f. sp. *cubense* race 1 tolerant cultivars and hybrids of bananas (Kavino et al., 2009). Previous reports by Migheli et al. (1996) on the biocontrol of *P. debaryanum* by *Arthrobacter* spp. elucidated the role of glucanase and protease in the lysis of mycelium.

The genome of *T. reesei* has been reported to possess genes encoding for 2 cellobiohydrolases, 5 endo-ß-1,4-glucanases, and different isoforms of ß-glucosidases, hemicellulases, and accessory enzymes (Martinez et al., 2008; Hakkinen et al., 2012; Dita et al., 2018). *CBH* encodes for exo-cellobiohydrolases or exoglucanases which are involved in the hydrolysis of 1,4-beta-D-glucosidic bonds in the external structure of cellulose and aid in cell wall degradation (Antonieto et al., 2014; Nogueira et al., 2015). CBH1 is reported to be an inducible gene in the presence of inducers, such as cellulose, sephorose, lactose, and cellobiose (Dos Santos et al., 2014; Nogueira et al., 2015); which was confirmed in the present study as pathogen interaction with CSR-T-3 induced the expression. On the contrary, endo-β-1,6-glucanase is one of the important genes for mycoparastic activity of *Trichoderma* sp which catalyzes cell wall degradation by breaking β-1,6 linkages internally (De la Cruz et al., 1995; Gruber and Seidl-Seiboth, 2011). In the present study, the upregulation of *CBH1*/*CBH2, EGL1*, and *XYN1* genes in TFTR have ascertained the mycoparasitism activity of CSR-T-3. Other signal transduction pathway genes were also found to be upregulated in the treatments involving CSR-T-3. The *TGA1* gene encodes for a G protein α subunit, which is involved in mycoparasitic coiling, conidiation (Rocha-Ramirez et al., 2002), formation of chitinase, and the production of antifungal metabolites (Reithner et al., 2005). *VEL1* controls the regulation of the pheromone system, mating partner sensing, and sexual and asexual development (Bazafkan et al., 2015). The *VEL1* gene encodes for velvet protein 1, a part of the VELVET complex composed of *LAE1, VEL1*/*VELA*, and *VEL2*/*VELB* proteins. This *VEL1* protein is also responsible for morphogenetic traits, is a key regulator of biocontrol in *T. virens*, and is highly conserved in different species of *Trichoderma* (Supplementary Figure 2) (Mukherjee and Kenerley, 2010). It is also reported to have a role in the induction of cellulase gene family expression which includes cellobiohydrolases, glucanases, chitinases, and xylanases (Karimi Aghcheh et al., 2016). The *TMK*1 gene encodes for a mitogen activated protein kinase (MAPK) which plays an important role in mycoparasitism by the regulation of mycoparasitic host attack, formation of aerial hyphae, and conidiation (Reithner et al., 2007; Schmoll, 2008; Swain and Mukherjee, 2020). Generally, MAPK are involved in signal transduction pathways that converge into the accumulation of secondary metabolites. Earlier, Reithner et al. (2007) reported that tmk1 deletion caused reduced mycoparasitic activity against *Rhizoctonia solani* and *Botrytis cinerea* and were also associated with the regulation of production 6-pentyl-a-pyrone and peptaibol antibiotics. The upregulation of the *TGA1, TMK1*, and *VEL1* genes indicate the possibility of enhanced chitinase activity. This information also supports the result of high chitinase and phenol activities in TFTR and TTR treatments in the study.

Biocontrol mechanisms of CSR-T-3 (*T. reesei*) against Fusarium wilt pathogen Foc TR4 (CSR-F-1) are considered to be mainly because of mycoparasitism, the production of antifungal metabolites, such as iturin C, ß-carophyllene etc., and a reduction in the fungal toxins like fusaristatin A, fusarin C, beauveric acid, and chlamydosporal production (Figure 6). This well-established concept of biocontrol reported to occur in *Trichoderma* species was confirmed by gene expression profiling of the fusaric acid biosynthesis and cellulolytic enzymes of *Fusarium* species and *T. reesei*, respectively. *Trichoderma* species colonize the epidermal root surface and also induce the plant to produce secondary metabolites that include compounds with low molecular weight inducing the expression of genes involved in the defense response against the pathogen (Hermosa et al., 2012; Malmierca et al., 2014; Sood et al., 2020). Earlier whole genome transcriptome analysis indicated that defense genes associated with CEBiP, BAK1, NB-LRR proteins, PR proteins, transcription factor, and cell wall lignification were expressed strongly in resistant variety Yueyoukang 1 indicating that these genes play important roles against Foc TR4 infection in bananas (Bai et al., 2013).

Developing a bioformulation using the CSR-T-3 *T. reesei* isolate based on the antagonistic effect on the pathogen and the epidemiology of the disease in the hotspot region, we standardized the application schedule and mode of application. A field evaluation of CSR-T-3 bioformulation in the sick plots of a hot spot area has clearly demonstrated the successful control of the pathogen which is evident from the reduced disease incidence at the same time contributed to the successful management of the disease. Earlier, Damodaran et al. (2019b) demonstrated successful control of Foc TR4 using ICAR-FUSICONT. To date, no product is available in India for biological control of Fusarium wilt disease Foc TR4 and this study is the first of its kind to find evidence for successful management.

## Conclusion

The current gaps in devising management schedules for Fusarium wilt disease include the scarcity of resistant varieties, no effective chemical control measure, the lack of a virulent biological control agent that could work at field level, and also their clear mode of action. Our study has been a solution to the latter category of biological control of Fusarium wilt disease of bananas caused by Foc TR4. We report here an effective virulent CSR-T-3 isolate of *T. reesei* that was successfully proven in a pot culture experiment and field evaluation in sick fields of hot spot areas of plants affected by Foc TR4. This biocontrol agent has been proven to have a good effect on the fruit yield and weight besides the suppression of the pathogen. The mechanism of mycoparasitism and reduction in toxin accumulation was also elucidated using gene expression and LC-MS analysis which has established the role of cell wall degrading enzymes. Signal transduction pathways inducing secondary metabolites with antifungal properties in *Trichoderma* has resulted in the suppression of toxins produced by Foc TR4 and thus conferred induced tolerance to banana plants. Field evaluation of the CSR-T-3 showed the potential of this bioagent in the management of the banana Fusarium wilt disease on a large scale. This study is a holistic approach to the technological advancement of *T. reesei* from lab to field in the management of Fusarium disease in bananas.

## Data Availability Statement

All raw data analyzed supporting this article will be made available by the authors. Public domain genome sequence data sets were used for primer designing and the accession numbers are provided in the article.

## Author Contributions

TD conceived, planned, and designed the experiments. SR designed the statistical methodology and analyzed the data. MM designed the gene expression studies, designed primers for expression analysis, and contributed equally to the manuscript preparation with TD. RG and KY performed the experiments of dual culture, pot, and field experiment. SK performed the molecular wet lab studies. IA performed the molecular identification of isolates. NK assisted in the disease screening experiments. SKJ and VKM performed the soil analysis of the pot soil used in the screening trial. All authors contributed to the article and approved the submitted version.

## Conflict of Interest

The authors declare that the research was conducted in the absence of any commercial or financial relationships that could be construed as a potential conflict of interest.
