# Supplementary material for: Biological Management of Banana Fusarium Wilt Caused by Fusarium oxysporum f. sp. cubense Tropical Race 4 Using Antagonistic Fungal Isolate CSR-T-3 (Trichoderma reesei)
Source: Front Microbiol. 2020 Dec 16;11:595845. doi: 10.3389/fmicb.2020.595845 (PMC7772460; doi:10.3389/fmicb.2020.595845)
Supplement: Supplementary file 1 [file Data_Sheet_1.docx]

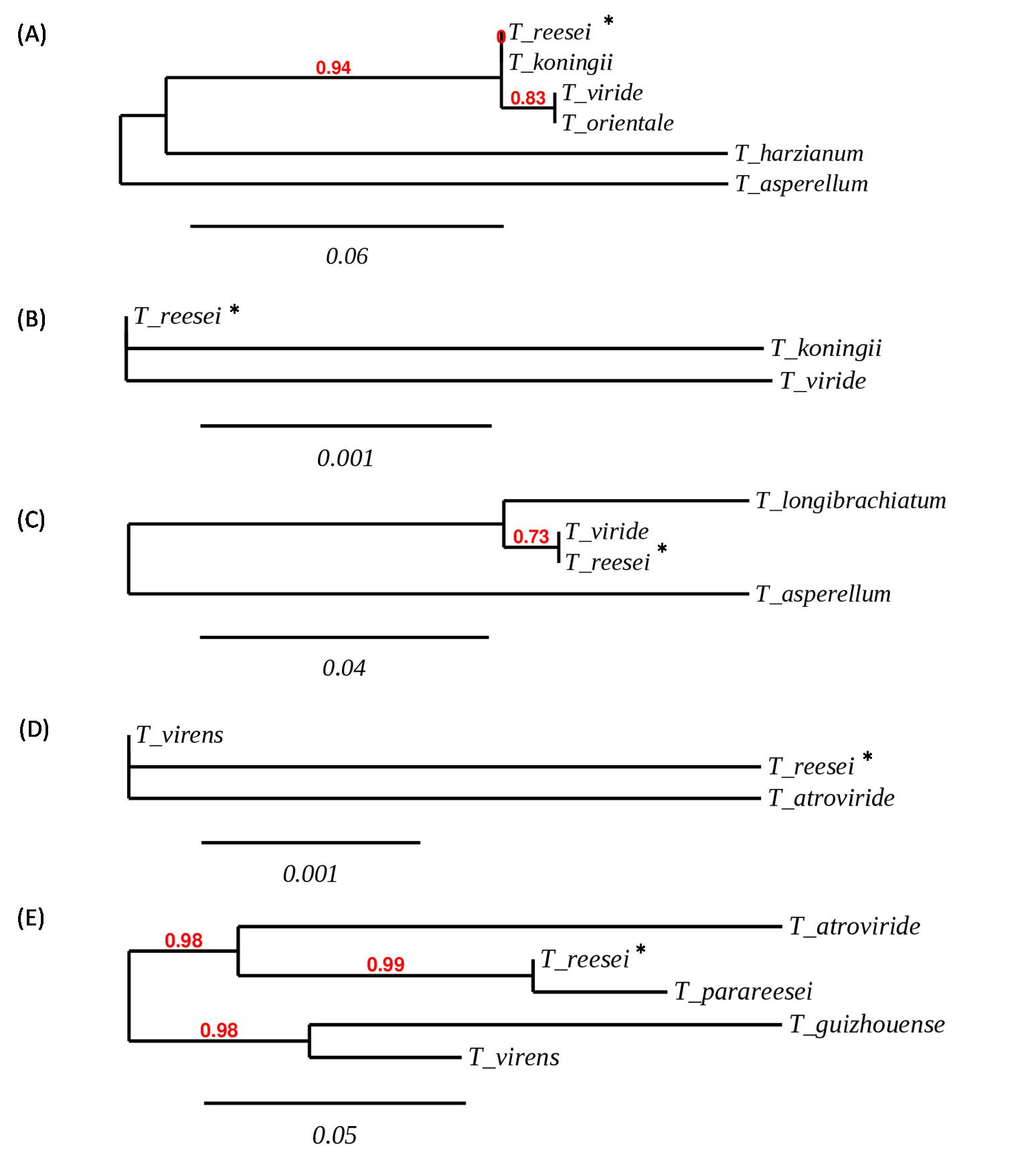


**Figure S1. Phylogenetic analysis showing the uniqueness and distinctness in the protein sequences of mycoparasitism and signal transduction genes of *Trichoderma reesei* with other *Trichoderma* species.** (A) *CBH1*, (B) *CBH2*, (C) *EGL1*, (D) *TMK1*, and (E) *VEL1*.


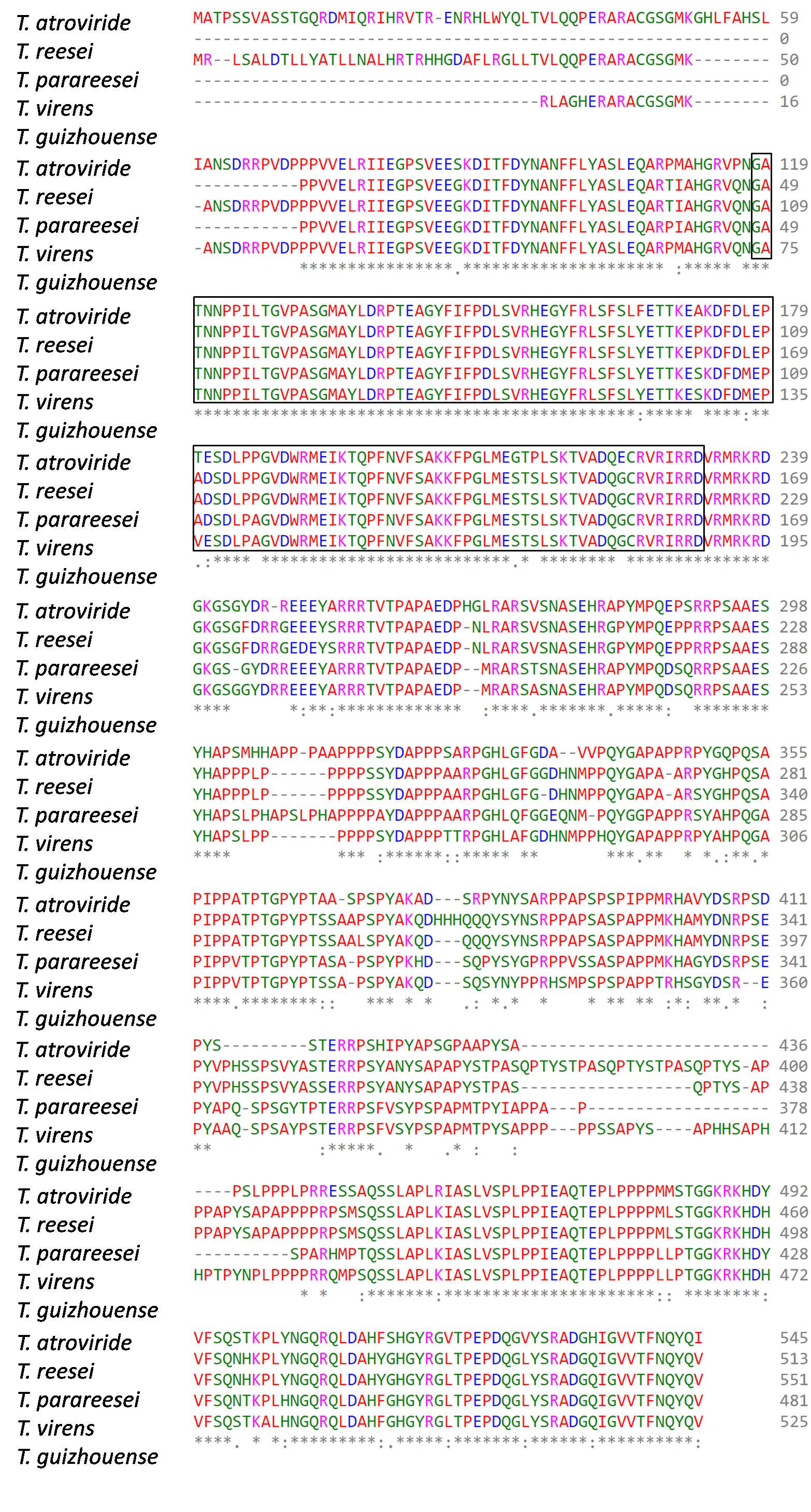


**Figure S2. Conserved domains in the *VEL1* gene product (VELVET1 protein) among different *Trichoderma* species.** The multiple sequence alignment of amino acid sequences of VEL1 protein shows conserved velvet domain (marked in box) among *Trichoderma* species.

**
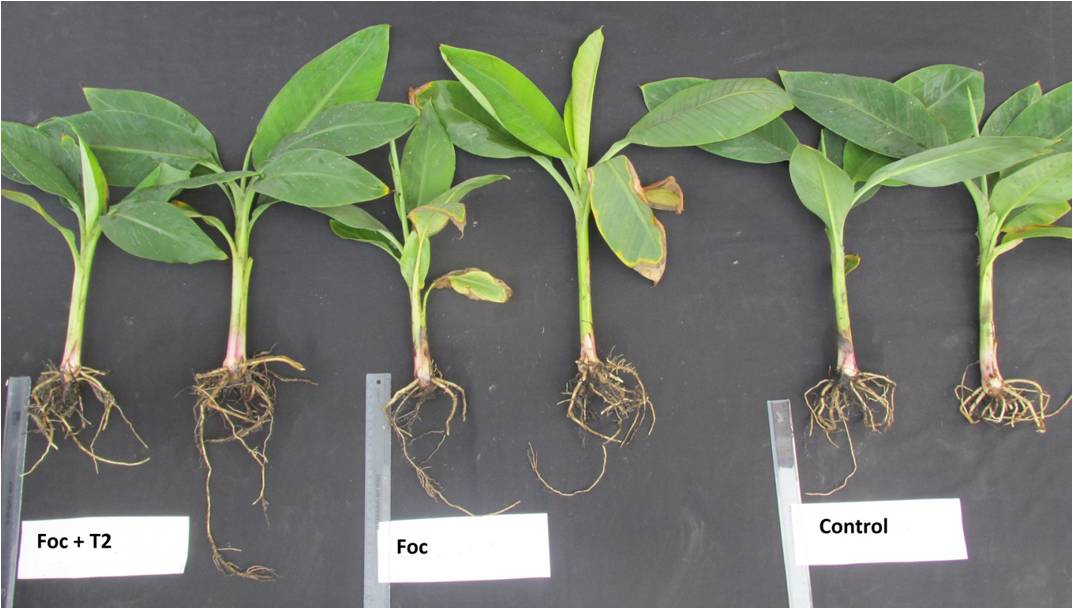
**

**Figure S3. Differences in the root characters of banana plants observed in TC, TF and TFTR treatments.** Rooting is affected in TF treatment while growth promotion is achieved in TFTR.
